# A systematic review of the psychosocial difficulties relevant to patients with migraine

**DOI:** 10.1007/s10194-012-0482-1

**Published:** 2012-09-23

**Authors:** Alberto Raggi, Ambra Mara Giovannetti, Rui Quintas, Domenico D’Amico, Alarcos Cieza, Carla Sabariego, Jerome Edmound Bickenbach, Matilde Leonardi

**Affiliations:** 1 Neurology, Public Health and Disability Unit, Neurological Institute C. Besta IRCCS Foundation, Milan, Italy; 2 Headaches Center, Neurological Institute C. Besta IRCCS Foundation, Milan, Italy; 3 Department of Medical Informatics, Biometry and Epidemiology (IBE), Ludwig-Maximilians-University, Munich, Germany; 4 Swiss Paraplegic Research Centre, Nottwil, Switzerland; 5 Faculty of Social and Human Sciences, School of Psychology, University of Southampton, Southampton, UK; 6 Disability Policy Unit, Swiss Paraplegic Research Centre, Nottwil, Switzerland

**Keywords:** Migraine, Disability, Preventive treatment, Symptomatic treatment, Pain, Headaches frequency

## Abstract

Migraine is a common disease which causes significant burden to individuals, in terms of personal suffering and activity reduction, and to societies, in terms of disease cost. The purpose of this study is to identify the most relevant psychosocial difficulties related to migraine, the variables associated with them and the most relevant determinants of their evolution over time. MEDLINE and PsychINFO were searched for studies published in English between 2000 and 2010 that examined psychosocial difficulties in persons with migraine with and without aura, from clinical trials and observational studies. Information on the description of each difficulty, its determinants of onset and change over time and associated variables were extracted and categorized at a higher level. In total, 34 difficulties have been collected from 51 papers: the most frequent were reduced vitality and fatigue, emotional problems, pain, difficulties at work, general physical and mental health, social functioning and global disability. Evidence exists that pharmacological treatments have an impact toward improvement in patients’ difficulties, in particular emotional problems, physical and mental health, difficulties with employment and global disability. Migraine treatments and decreased headaches frequency are the major determinants of improvements in psychosocial difficulties, while no information is available for determinants of worsening; understanding the role of such factors is of primary public health relevance, given the high prevalence and the relevant personal and societal costs of migraine.

**Electronic supplementary material:**

The online version of this article (doi:10.1007/s10194-012-0482-1) contains supplementary material, which is available to authorized users.

## Introduction

Migraine prevalence is around 15 % (17.6 % in women) in European Countries [1, 2], and its average annual cost per patient is estimated at 1,222€, of which 93 % are indirect costs related to reduced productivity and absenteeism [3]. Migraine adversely affects patients’ health-related quality of life (HRQoL), independently from comorbidities such as mood or anxiety disorders [4–7], and contributes to several difficulties in daily life. Because of this, the World Health Organization (WHO) recognizes migraine as a high priority public health problem [8].

The impact of migraine on patients’ daily life has been assessed using measures of disability, activity limitation or HRQoL, often as secondary measures of treatment outcome [4–7, 9, 10]. Disability associated with migraine is strictly related to its severity: areas of functioning such as communication, mobility, self-care, participation in society, relationships with others [11] and with family members [12] are particularly affected. Yet, the difficulties experienced by patients have never been systematically described in a literature review that looks at their impact both at the personal and societal levels. To our knowledge, only factors related to gender differences have been analyzed in a review [13]: there the authors concluded that gender and social role expectations, as well as coping strategies, are different and this determines differences in response to pain. However, no information on course and determinants of these difficulties was included in this review, which is an important gap if we are to reduce the indirect effects of migraine on patients’ daily lives.

We therefore propose a systematic literature review of the psychosocial difficulties (PSDs) associated with migraine. For our purposes, PSDs are understood in terms of the biopsychosocial model found in the International Classification of Functioning, Disability and Health (ICF) [14]. According to this model, PSDs are impairments of mental functions and activity limitations and participation restrictions that involve social interactions, such as in work, family life and leisure activities, as well as daily activities such as those connected to daily routing, homework or mobility. Since these difficulties account for the personal and socio-economic burden of migraine, it is important to identify and understand the impact of those factors that are responsible for the onset and course of PSDs.

The aims of this review are twofold. First, to systematically identify the range of PSDs reported in the literature on migraine; second, to identify the most relevant determinants of onset and change over time for PSDs, as well as the variables that are associated with these PSDs. Since PSDs are defined according to the ICF’s biopsychosocial model, the literature review will be organized according to the ICF classification structure.

## Methods

### Search strategy

MEDLINE and PsychINFO were searched for studies published in English between January 2000 and May 2010 that examined PSDs in persons with migraine. Ten years enabled us to focus on current treatment strategies—i.e., the established use of triptans and of preventive medications—which have had an impact on PSDs, and to find studies published during a period in which the issue of the burden of brain disorders is topical.

Search terms were customized to each database by combining the term migraine with the following key words: psychosocial*, Quality of Life/, Personal Satisfaction/, exp Human Activities/and exp Social Support/disabilit*, homelessness, environmental factor*, exp Interpersonal Relations/, Quality of Life/, Personal Satisfaction/, exp Human Activities/, paternalism/, prejudice/, psychosocial deprivation/, social values/, exp Social Problems/, Social Adjustment/, social isolation/, stereotyping/, exp Social Environment/, exp emotions/, exp family/, exp socioeconomic factors/, exp life style/, exp Disability evaluation/, Communication Barriers/, Adaptation, Psychological/, Aggression/, Psychological stress/, (community not microbial community), or (sexual* or intimacy).

### Paper inclusion and exclusion criteria

Studies were included if patients were diagnosed with migraine with or without aura, according to the criteria of the International Headache Society’s classification, first [15] or second [1] edition. Studies were excluded if at least 50 % of patients had comorbidities for substance abuse, epilepsy, secondary headaches, cerebrovascular diseases or reported more than 15 headaches/month.

Journal articles in English reporting randomized controlled trials, controlled clinical trials, observational studies, and economic evaluation studies were included. In case of multiple publications dealing with the same data, the paper published in the journal with the highest impact factor was included. Papers were excluded if they were primary prevention studies, phase I and II studies, ecologic studies, systematic reviews, case report/case series, qualitative studies and psychometric studies (development or validation of questionnaires or scales), commentaries, letters to the editors, editorials and conference reports. Since we were particularly looking for determinants of PSDs over time, longitudinal designs were of primary interest. Cross-sectional studies were included if the content of the paper was judged to be of primary importance for the identification of relevant PSD or their associated variables. Other exclusion criteria were the absence of psychosocial factors, focus on caregivers’ burden, focus on risk factors leading to migraine and not to PSD.

### Paper selection and data extraction

Abstracts of papers that were selected from database searches were screened by two experienced researchers (AR and AMG). To insure quality and consistency of data extraction, 20 % of the abstracts were randomly selected for a second check by another reviewer (RQ) who was blinded to the decisions of the first two. Each reviewer had to rate the paper as excluded, eligible or ambiguous. Full texts of papers that were judged eligible or ambiguous were then analyzed, and 10 % of the full texts were double checked by two reviewers independently. An evaluation of the paper’s quality was performed using the National Institute for Health and Clinical Excellence guidelines [16], and the quality of the study was judged poor (1), acceptable (2), good (3) or excellent (4): papers with poor quality were excluded. For practical reasons, quality scores will be reported at a group level only.

Extracted information was referred to the description of each PSD, the determinants of their onset or change over time, as well as other variables associated with PSDs. Determinants of change over time were extracted exclusively from longitudinal studies; variables associated with PSDs were collected from cross-sectional studies or from cross-sectional analysis in longitudinal studies. Information on study design, type of intervention and characteristics of the study population were extracted as well.

Information synthesis was a three-step process. First, collected PSDs—extracted exactly as they were presented in each paper—were grouped into categories based on the ICF classification according to standardized ICF linking rules [17]. ICF linking is an established procedure requiring the content of items in assessment instruments to be connected to the most precise ICF category possible: training was provided for this procedure and 10 % of full-text were double checked with regard to linking results. The ICF categories representing the PSDs were then grouped by similarity of content into overarching categories according to Popay’s guidelines [18] on how to analyze narrative reviews. Finally, following a methodology recently employed by Cabello et al. [19], evidence was judged as strong if there were at least two good papers reporting the same results; limited if there was only one good paper and some acceptable studies reporting similar result; controversial in case studies reported contrasting results.

## Results

**Fig. 1:**
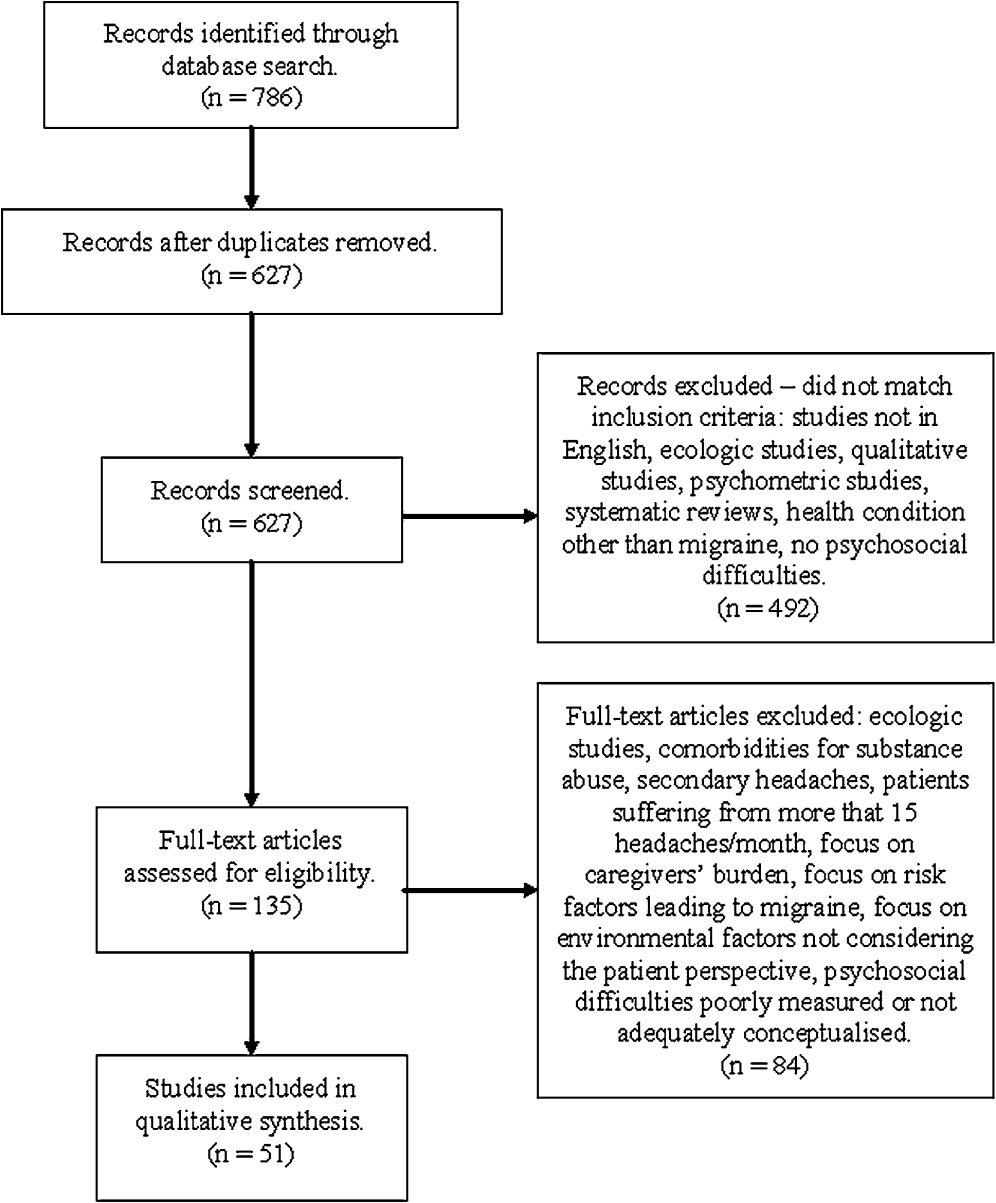
Flowchart of paper selection

In total, 627 abstracts were screened (Fig. 1): 492 records were excluded at abstract screening, mainly because they did not include a measure of PSD consistent with selection criteria or because the main health condition was not migraine. Therefore, 136 full texts were read, after which 84 papers were excluded: these exclusions were mainly due to a poor measurement or conceptualization of PSDs, inadequacy of research design or the presence of comorbidities. Therefore, 51 papers were included in the synthesis [20–70].

Table 1 reports the major characteristics of included studies. Sample sizes were heterogeneous and varied between 12 and 5,417. Percentage of females was reported in 46 studies and ranged between 43.8 and 100 %, with a mean of 80.2 %. Information about age was reported in 48 papers and ranged between 12 and 88 years, with an aggregate mean age of 40.6 years. Disease duration, reported either as years from the onset of migraine or years since diagnosis was reported in 14 studies, with a mean of 13.0 years. The mean quality of studies was 2.7, median 3, i.e., generally good.

**Table 1:** General characteristics of included studies

	Observational studies		Clinical trials			Total studies included (no. 51)
	Cross-sectional studies (no. 9)	Longitudinal studies (no. 12)	Case–control studies (no. 2)	Controlled trials (no. 5)	Randomized trials (no. 23)	
Total sample size	5,155	7,147	471	1,051	7,004	20,852
Mean ± SD	572.8 ± 841.1	595.6 ± 1,526.8	235.5 ± 191.6	210.2 ± 350.4	304.5 ± 358.1	401.0 ± 839.7
Min.–max.	28–2,245	20–5,471	100–371	20–835	12–1,506	12–5,471
Females (%)	82.2	80.7	84.7	86.1	77.5	80.2
Mean age (years)	41.1	41.2	36.9	39.6	39.9	40.6
Mean disease duration	13.6	12.1	18.9	–	18.2	13.0
Diagnosis						
Migraine with aura (%)	4	5.1	14	29.6	26.2	9.8
Migraine without aura (%)	96	94.9	86	70.4	73.8	90.2
Overall quality (mean ± SD)	2.4 ± 0.5	2.4 ± 0.5	3 ± 0	2.4 ± 0.5	2.9 ± 0.7	2.7 ± 0.7

Percentages, mean age, mean disease duration were calculated in reference to the number of patients enrolled for each study type

Table 2 reports the span of PSDs found in included studies: in total, 34 different PSDs have been collected and these have been reported 231 times, mostly from randomized trials (91 times) and longitudinal observational studies (63 times). Due to the amount of PSDs collected, only those addressed at least ten times are described in detail. These were problems with energy and drive function, emotional functions and sensation of pain; difficulties with remunerative employment; general evaluations of mental health and physical health, social functioning and global disability evaluations.

**Table 2:** Psycho-social difficulties identified in included studies categorized according to ICF structure

ICF category	Specific PSD	Observational studies		Clinical trials			Total studies included (no. 51)
		Cross-sectional studies (no. 9)	Longitudinal studies (no. 12)	Case–Control studies (no. 2)	Controlled trials (no. 5)	Randomized trials (no. 23)	
B130: Energy and drive functions	Vitality	2 (4.3 %)	5 (7.9 %)	–	1 (5 %)	4 (4.4 %)	12 (5.2 %)
	Fatigue	1 (2.1 %)	1 (1.6 %)	–	–	1 (1.1 %)	3 (1.3 %)
	Motivation	–	1 (1.6 %)	–	–	–	1 (0.4 %)
B134: Sleep functions	Sleep	1 (2.1 %)	–	1 (10 %)	–	3 (3.3 %)	5 (2.2 %)
B140: Attention functions	Cognitive functions: attention	2 (4.3 %)	–	–	–	1 (1.1 %)	3 (1.3 %)
B144: Memory functions	Cognitive functions: memory	1 (2.1 %)	–	–	–	–	1 (0.4 %)
B152: Emotional functions	General emotional functions	2 (4.3 %)	3 (4.8 %)	–	1 (5 %)	7 (7.7 %)	13 (5.6 %)
	Depressive mood and symptoms	4 (8.5 %)	4 (6.3 %)	2 (20 %)	1 (5 %)	5 (5.5 %)	16 (6.9 %)
	State anxiety	5 (10.4 %)	4 (6.3 %)	1 (10 %)	1 (5 %)	5 (5.5 %)	16 (6.9 %)
	Trait anxiety	–	–	1 (10 %)	–	–	1 (0.4 %)
	Stress	1 (2.1 %)	2 (3.2 %)	–	–	2 (2.2 %)	5 (2.2 %)
	Anger	1 (2.1 %)	1 (1.6 %)	–	–	1 (1.1 %)	3 (1.3 %)
B156: Perceptual functions	Perceptual functions	2 (4.3 %)	–	–	–	–	2 (0.9 %)
B280: Sensation of pain	Pain	2 (4.3 %)	4 (6.3 %)	–	1 (5 %)	4 (4.4 %)	11 (4.7 %)
D230: Carrying out daily routine	Carrying out daily routine	–	–	–	–	1 (1.1 %)	1 (0.4 %)
D240: Handling stress of other psychological demands	Coping strategies	1 (2.1 %)	1 (1.6 %)	–	1 (5 %)	2 (2.2 %)	5 (2.2 %)
D4: Mobility	General mobility	–	–	–	–	3 (3.3 %)	3 (1.3 %)
D640: Doing housework	Doing housework	1 (2.1 %)	1 (1.6 %)	1 (10 %)	–	1 (1.1 %)	4 (1.7 %)
D7: Interpersonal interactions and relationships	Relationships with others in general	1 (2.1 %)	–	–	1 (5 %)	2 (2.2 %)	4 (1.7 %)
D740: Formal relationships	Relationships with health professionals	1 (2.1 %)	–	–	–	–	1 (0.4 %)
D760: Family relationships	Family relationships	1 (2.1 %)	–	–	–	–	1 (0.4 %)
D850: Remunerative employment	Time restriction	2 (4.3 %)	1 (1.6 %)	1 (10 %)	–	3 (3.3 %)	7 (3.0 %)
	Reduced efficiency	1 (2.1 %)	2 (3.2 %)	–	1 (5 %)	5 (5.5 %)	9 (3.9 %)
D920: Recreation and leisure	Leisure activities	2 (4.3 %)	2 (3.2 %)	1 (10 %)	–	2 (2.2 %)	7 (3.0 %)
Overall scores: linking to ICF categories not determined	Mental health	2 (4.3 %)	5 (7.9 %)	1 (10 %)	2 (10 %)	5 (5.5 %)	15 (6.5 %)
	Physical health	2 (4.3 %)	4 (6.3 %)	1 (10 %)	2 (10 %)	6 (6.6 %)	15 (6.5 %)
	General health	2 (4.3 %)	3 (4.8 %)	–	–	1 (1.1 %)	6 (2.6 %)
	Global functioning or global disability	3 (6.4 %)	5 (7.9 %)	–	3 (15 %)	15 (16.5 %)	26 (11.2 %)
	Social functioning	2 (4.3 %)	4 (6.3 %)	–	1 (5 %)	3 (3.3 %)	10 (4.3 %)
	Quality of life	–	3 (4.8 %)	–	1 (5 %)	4 (4.4 %)	8 (3.5 %)
	Role emotional	2 (4.3 %)	4 (6.3 %)	–	1 (5 %)	1 (1.1 %)	8 (3.5 %)
Personal factors: not linkable to ICF categories	Self-efficacy	–	1 (1.6 %)	–	1 (5 %)	1 (1.1 %)	3 (1.3 %)
	Locus of control	–	1 (1.6 %)	–	1 (5 %)	–	2 (0.9 %)
	Satisfaction with specific life domains	–	1 (1.6 %)	–	–	3 (3.3 %)	4 (1.7 %)
Total number of PSD		47 (100 %)	63 (100 %)	10 (100 %)	20 (100 %)	91 (100 %)	231 (100 %)

Table 3 reports information on the determinants of course over time and the variables associated with the most frequently addressed PSDs. Variables related to mental functions were generally reported as associated with PSDs, and in some cases these variables are PSDs themselves. With regard to the determinants of change over time, only determinants of improvement were found: most of the determinants referred to treatments, duration and frequency of headaches and the presence of pain. Pain was also found to be a determinant of PSD onset in two studies: in the first, it was found to be a determinant of fatigue and problems with perceptual and cognitive functions [20]; in the second, it was found to be a determinant of difficulties with employment, both reduced efficiency and reduction in time [21].

**Table 3:** Variables reported as associated and determinants of improvement of the most frequently addressed PSDs

Related variables	PSD linked to ICF categories				PSD with linking to ICF not determined			
	B130: Energy and drive functions	B152: Emotional functions	B280: Sensation of pain	D850: Remunerative employment	Mental Health	Physical Health	Social functioning	Global functioning or disability
Mental functions								
Depressive mood and symptoms					A (1)	A (1)		
General emotional functions	A (1)				A (1)		A (1)	
Energy and drive		A (4)						
Sleep		A (2)						A (1)
Stress		D+ (2)						
Pain	A (3) D+ (2)^a^	A (1) D+ (2)		A (1) D+ (2)^b^		D+ (1)	A (1) D+ (1)	D+ (1)
Self-efficacy		D+ (2)						D+ (1)
Reduced efficiency at work	A (1)							
Reduced participation to social activities		A (1)						
Issues related to migraine								
Duration	A (1)							
Frequency	A (1) D+ (3)	A (2) D+ (4)	D+ (2)	D+ (1)	D+ (2)	A (1) D+ (3)	D+ (2)	D+ (3)
Being aware of migraine		A (2)						A (1)
Treatment								
Symptomatic	D+ (2)	D+ (3)	D+ (2)	D+ (4)	D+ (1)	D+ (1)	D+ (2)	D+ (5)
Prophylactic	D+ (2)	D+ (5)	D+ (2)	D+ (2)	D+ (2)	D+ (4)	D+ (2)	D+ (6)
Complementary/alternative		D+ (3)	D+ (1)					
Psychological therapy								D+ (1)
Surgery		D+ (1)			D+ (2)	D+ (2)		D+ (3)
Multidisciplinary care		D+ (1)	D+ (1)					D+ (1)
Overall quality of life		A (1)		A (1)			A (1)	
General health	A (2)	A (3)		D+ (1)				
Global functioning or disability	A (2)	A (1)	A (1)	A (1)	A (1)	A (1)	A (1)	
Passage of time		D+ (1)						D+ (1)

The number between brackets indicates the frequency of determinants and associated variables

A Variables associated with PSDs; D+ variables acting as determinants of improvements of PSDs

^a^Pain was reported also as a determinant of onset of problems with fatigue in one study

^b^Pain was reported also as a determinant of onset of difficulties with remunerative employments—reduced efficiency and time restriction—in one study

### Energy and drive functions

This set of functions includes in particular fatigue and vitality, which represent 6.9 % of all PSDs. There is strong evidence of the association of fatigue and headache pain [20, 22], while the evidence is limited for the association between decreased motivation and headache duration [22], and between reduced vitality, headache frequency [23], general emotional problems [24] and reduced work efficiency [20]. Limited evidence also exists for the association between reduced vitality and fatigue, low general health [22] and increased disability [25, 26].

Strong evidence exists that pain reduction [27, 28] and decreased headache frequency [20, 29, 30] positively affect patients’ vitality. Limited evidence exists that symptomatic medications, such as Almotriptan [26] and Rizatriptan [31], improve patient vitality. Limited evidence also exists about the utility of Topiramate in improving vitality [32] and reducing fatigue [33] as well as of other prophylactic agents, including antiepileptics, antidepressants, neuroleptics, and beta-blockers in improving vitality [29].

In sum, there is strong evidence that problems with fatigue and reduced vitality were associated with the presence of pain and that pain reduction and decreased headache frequency determine improvement in vitality, while there is limited evidence for the association between vitality, work efficiency, general health and disability. The evidence of prophylactic and symptomatic medications effect toward the improvement of vitality and fatigue reduction is limited.

### Emotional functions

A relevant part of identified PSDs refers to emotional problems, in particular anxiety and depressive mood, as well as anger, stress, concerns about the disease, sense of inadequacy and fear of migraine attacks. Taken as a whole, these issues represent 23.3 % of all PSDs, with anxiety and depressive mood being the most commonly addressed (6.9 % each).

Limited evidence exists concerning the association between anxiety and depressive mood and sleep problems [34], the association between depressive mood, anger, stress and anxiety, and between reduced vitality, fatigue, patient general health state and reduced participation to social activities [22]. In a population study, persons with migraine had higher depression and anxiety compared to healthy subjects: there is limited evidence that patients who were aware of their condition reported slightly better scores [35]. Limited evidence exists on the association between migraine frequency and anxiety [36], while there is strong evidence that general emotional problems are associated with increased disability [26] and reduced HRQoL [37].

Limited evidence exists about the positive impact of stress reduction and improvement of depressive mood and anxiety [38], while strong evidence exists on the effect of pain reduction toward improvement in general emotional problems [27] and anxiety [39]. Limited evidence exists about the role of migraine frequency: patients with less frequent headaches reported lower anxiety levels [40], and those who underwent a reduction of headache frequency had an improvement in anxiety and mood level [41, 42], also in association with a good sense of self-efficacy [41]. Consistently strong evidence was found for the positive effect of prophylactic and symptomatic medications. Prophylactic therapies, such as Topiramate [32, 43], Amitriptyline [43] and Botulinum Toxin type A [33], or symptomatic therapies such as Sumatriptan [22, 42] or Almotriptan [26], determine a reduction of headache frequency and intensity but also provide beneficial effects on the reduction of emotional problems associated with migraine, in particular low mood and anxiety [33]. Two studies report limited evidence that complementary non-medical treatments, such as massage therapy [44] and yoga [45], determine a reduction of anxiety and mood problems.

In sum, there is limited evidence for the association of emotional problems, in particular anxiety and depressive mood and factors such as low vitality and fatigue. There is also evidence, although limited, that headache frequency decrease and complementary/alternative treatments have a direct positive effect on anxiety and mood. Finally, there is strong evidence that prophylactic and symptomatic medication have an impact on the reduction of emotional problems.

### Pain

Pain is the cardinal symptom of migraine but, despite this, it has been directly considered as a PSD in a relatively small number of paper (4.7 % of all PSDs), with a poor pattern of association to global disability [25].

Limited evidence exists about the positive impact of prophylactic medications, such as Topiramate [32] and Botulinum Toxin type A [33], and symptomatic medications, such as Rizatriptan [31], Sumatriptan and Naproxen Sodium [42] on pain reduction.

### Employment

Difficulties in employment represent 6.9 % of all PSDs and were mostly conceptualized as reduced efficiency and restriction in time devoted to work, i.e., partial absence or missed workdays.

Limited evidence exists on the association of time restriction with overall HRQoL [25], and with global disability and the presence of pain [26]. Limited evidence is available for the effect of pain reduction on improved work efficiency [27, 46], and of reduced headache frequency on decreased number of lost workdays [30]. Strong evidence exists on the effect of symptomatic medication such as Almotriptan [26], Rizatriptan [31], Sumatriptan [47] and Eletriptan [48] on improving work efficiency. Strong evidence also exists on the effect of prophylactic medications, such as Botulinum Toxin type A and improved efficiency [33], as well as of Topiramate/Amitriptyline and reduction of missed workdays [43].

In sum, problems in employment represent a difficulty both because of reduction in efficiency and restriction in time. The pattern of association is scarcely determined, while reduction of pain and prophylactic and symptomatic medications was reported as a determinant of improvement in workplace efficiency and reduction of missed workdays.

### General physical and mental health

General descriptions of mental and physical health constitute 6.5 % of all PSDs each, share a similar pattern of association and are also affected by the same determinants of improvement.

Limited evidence exists that poor mental health is associated with general emotional problems [24] and that both mental and physical health are associated with depressive mood [37] and overall disability [25]. Limited evidence also exists that low physical health is associated with headache frequency [23].

Strong evidence exists that mental and physical health improve consistently with reduction of headache frequency [23, 29, 30], while the evidence of improvement in physical health as a consequence of pain reduction is limited [28]. There is also limited evidence that symptomatic treatment with Rizatriptan is effective in improving both physical and mental health [31]. With regard to prophylactic treatment, strong evidence exists that Topiramate is effective in improving both mental and physical health [24, 32, 49], while the evidence of the effect of beta-blockers such as Nebivolol and Metoprolol in improving physical health is limited [50]. Improvement in both physical and mental health after a surgical approach (deactivation of peripheral migraine headache triggers) was documented in two studies, but the evidence is limited [51, 52].

In sum, limited evidence exists that physical and mental health are associated with emotional problems, headache frequency and to overall disability. Strong evidence exists that prophylactic treatment with Topiramate and reduced headache frequency improve physical and mental health, while the evidence of the effect of pain reduction, utilization of Rizatriptan, beta-blockers as well as surgical approaches is limited.

### Social functioning

Issues of social functioning, in general, represent 4.3 % of all PSDs. Problems in this area have been weakly associated with general emotional problems [24], HRQoL [25], disability and the presence of pain [26].

Limited evidence exists about the positive effect of pain reduction [28] and decreased headache frequency [29, 30] in improving social functioning. Consistent but limited evidence also exists about the effect of both symptomatic, such as Almotriptan [26] and Rizatriptan [31], and prophylactic medication, such as Topiramate [32] and Botulinum toxin type A [33] to improve social functioning.

### Global disability

The concept of global disability implicitly encompasses all the PSDs a person may experience in relation to migraine. PSDs were frequently reported at this global level (in 11.2 % of the cases), which is the single most frequently reported category.

Limited evidence exists that global disability is associated with poor subjective sleep quality [34] and with self-awareness of migraine [35].

Limited evidence exists that patient disability is positively influenced by decreased headache frequency [23, 41, 53]. With regard to treatments, limited evidence exists that symptomatic medication, such as Sumatriptan [42, 47], Naproxen Sodium [42] Zolmitriptan [54] and Eletriptan [48] have an impact on disability reduction. Strong evidence is instead reported by studies documenting improvements due to prophylactic medication such as Topiramate [32, 43, 49, 55, 56], Botulinum Toxin type A [33], subcutaneous histamine [56], beta-blockers such as Nebivolol and Metoprolol [50] and Amitriptyline [43]. Limited evidence is available for the effect of pain reduction, achieved through physical exercise, on disability reduction [57]. There is limited evidence of the effect of surgical approaches—deactivation of peripheral migraine headache triggers [51, 52] and interatrial shunt closure [58]—on disability improvement. Limited evidence also exists that disability improves after the administration of a home-based behavioral training [59], and as a consequence of self-efficacy improvement, obtained with a self-administered behavioral intervention [41]. There is limited evidence that a multidisciplinary intervention aimed to reduce pain (sessions of exercise therapy, stress management sessions, relaxation therapy, dietary lectures and massage therapy sessions) was also effective in disability reduction [60]. Finally, limited evidence was found for the effect of passage of time [61].

In sum, general evaluation of disability was the most commonly evaluated PSDs category found in the present review. Reduction of disability in general is positively associated with the effectiveness of treatment: the evidence is limited for the effectiveness of symptomatic medications, behavioral interventions, surgical approaches and physical exercise, while it is stronger for the effectiveness of prophylactic medications.

## Discussion

This review offers an overview of the PSDs reported in the literature on migraine, the variables associated with them and the determinants that influence their onset and course. The most frequently studied PSDs were related to eight areas: emotional problems, reduced vitality and fatigue, pain, difficulties at work, reduced physical and reduced mental health, poor social functioning and increased global disability. A few variables were identified as associated with a handful of PSDs, namely global disability, emotional problems, pain and headaches frequency. On the contrary, there is more evidence that migraine-specific treatments improve emotional problems, physical and mental health, difficulties with employment and global disability. We found no studies evaluating possible determinants of worsening of PSDs and the presence of pain only was identified as a determinant of PSD onset.

To our knowledge, there is no previous attempt to systematically address PSDs relevant to migraine patients according to a definition based upon the ICF. A previous review [13] provided evidence that gender and social role expectations, as well as coping strategies, are different and this determines differences in response to pain. In our review, we expanded the scope of problems reported by migraineurs, and added information on the course and factors that influence the improvement of these difficulties. Our results also cast light on the conceptualization of disability found in migraine studies. The Migraine Disability Assessment Schedule (MIDAS) [71] is a reference point to assess disability in migraineurs and is used in the majority of publications dealing with outcome of migraine. However, it covers only a small part of the entire burden of living with migraine. A major result of our study was the focus on several areas that may be relevant to describe the problems experienced by patients with migraine, and that we believe should be investigated.

The determinants of improvement identified here can be roughly divided in two areas. The first includes variables referred to features of the disease itself, e.g., frequency of headaches and presence of pain. In general, limited evidence was found for the effect of these determinants, although they covered the full span of PSDs. The observed trend acknowledges that reduced headache frequency and pain decrease have a positive effect on improvement in vitality and fatigue—for which strong evidence was derived—emotional problems, physical and mental health, social functioning, work ability and global disability. The second area deals with prophylactic and symptomatic treatments. Studies on symptomatic medication herein included were focussed on different kinds of triptans rather than anti-inflammatory agents, and there is strong evidence that these medications determine an improvement in emotional problems and work efficiency. However, the most important determinants of PSD improvement found in this literature review were prophylactic medications, and there is strong evidence that these medications positively affect emotional problems, improve work efficiency, global disability, physical and mental health.

While several pharmacological studies have been published in the past years, limited data exist on non-pharmacological treatments. There is only sparse evidence on the effect of complementary treatment and psychological therapy: two studies showed efficacy of massage therapy and yoga for the improvement of anxiety and mood problems [44, 45], while one study found that home-based behavioral training might be effective to improve patient functioning [59]. The fact that such non-pharmacological interventions did not frequently occur in this literature review is in part due to the search strategy that gave primary relevance to longitudinal intervention studies, i.e., clinical trials of acute or prophylactic medication. Surgery is not a common procedure for migraine, rather it is indicated for the treatment of chronic cluster headache [72] and there are few experience on its use to treat patients with drug-resistant chronic migraine [73] also with comorbidity to depression [74]. As a consequence, the extent to which non-pharmacological treatments might improve migraineurs’ difficulties is still an open question that should be addressed in future research.

The articles included in the present review were mostly reports of clinical trials, which provided a strong control over study variables. In daily practice, however, clinicians have to deal with acceptance and adherence to treatment, which is a relevant issue for both prophylactic and acute migraine treatments [75–77]. Among migraineurs, the issue of non-adherence to treatment may have different implications, varying from inadequate timing in taking triptans, to not accepting prophylactic medications, to the overuse of symptomatic ones. Medication overuse, jointly with comorbidity to mood problems [78, 79], might determine worse health outcomes. In fact, problems with adherence to migraine treatment might be further on amplified by low treatment adherence which in mood disorders is around 40 % [80]. Multidisciplinary treatment has been proposed as a strategy for improving adherence to treatment, but results are conflicting [60, 81–83]. The implication of this is that our results mostly report facts that have been generated in the ideal contexts of clinical trial, but the situation of patients in real-life settings might be quite different.

Headache frequency is a determinant that deserves a separate comment. Frequency was found to be associated with the most relevant PSDs, but with strong evidence only for improvements in vitality, physical and mental health. It should be pointed out that reduction of headache frequency—which is not a PSD itself—is generally the primary endpoint (for e.g., in clinical trials on the use of prophylactic medications), while PSDs such as vitality were viewed as secondary outcome measures. Considering the aim and the methodology of the present review, the implication of this fact is that we are likely to underestimate the causal relationship between the treatment intervention tested in clinical studies, the magnitude of their effect on primary endpoints, and the improvements in PSDs that we focus in this review.

Some limitations of this study should be mentioned. Even though our search was extensive, we cannot be sure that all relevant articles were located. The fact that no determinant of PSD worsening was found is likely to be due to a publication bias, with studies that report negative results not being published. Some aspects of the included studies may have influenced our results, in particular the fact that there is an over-representation of data derived from the MIDAS [71] and the Short-Form 36 Health Survey (SF-36) [84]: taken together, they were used in 28 of the 51 included studies. The MIDAS may provide indications of either global disability or problems with work, household and leisure activities, which were herein described as separate PSDs. Similarly, SF-36-derived data were either reported as summary scores and therefore described as general health scores, or as subscales (e.g., vitality and social functioning) and thus reported as separate PSDs. Since these instruments are almost always used, the same PSDs are almost always reported, so that information about other PSDs are less frequently reported and remain almost unknown. However, the representation of PSDs associated with migraine is partial. The reason for this is that while there is a homogeneity due to the amount of data derived from MIDAS and SF-36, the number of PSDs is not describable in a synthetic way if the purpose is to avoid the reporting of known issues such as increased disability and reduced quality of life. With our synthesis, we tried to balance the opposite needs of being comprehensive and synthetic. Investigators in the field of migraine are therefore encouraged to evaluate other kinds of daily difficulties not included in commonly used instruments, and to include outcome measures that are able to capture the burden of migraine in a comprehensive way.

As we were interested to evaluate the course over time and determinants of PSDs’ course over time and the determinants of PSD change over time, we finally included several clinical trials: this is likely to reduce the ecological validity of our results, as subjects participating in clinical trials are exposed to a situation that is not the same commonly found in daily clinical practice.

A comment is also needed for pain, which was reported as a PSD in a relatively limited number of studies, e.g., when pain severity was directly assessed (for e.g., with the visual analog scale contained in MIDAS). In other studies, pain was considered to be a determinant, for example when reduction in pain was not directly measured, but was used to create groups of subjects for between-subjects analysis (e.g., the percentage of subjects achieving pain relief or experiencing pain reduction by a given timeframe following the intake of a medication).

Finally, the heterogeneity across studies should also be taken into account. The studies were very different with respect to sample size, number and duration of follow-up as well as study designs that provide different levels of control over confounding variables. Disease duration was reported in a small number of studies, hence preventing the evaluation of the effect of length of exposure to different PSDs, which may be influenced by disease duration.

## Conclusions

Our results confirm that migraine is a burdensome disease and that migraineurs experience several PSDs, in particular emotional problems, reduced vitality, pain, increased disability, difficulties with work, mental and physical health, social functioning. Our results also show that symptomatic and prophylactic treatments, by decreasing headache frequency and reducing pain, also determine a reduction in patient difficulties, thus reducing the burden associated with migraine. However, we know little about the factors that determine the worsening of PSDs, and understanding the role of these factors is essential for the development of prevention programs focusing on the PSDs associated with migraine. These actions might provide a wider understanding of the burden, personal and economic, associated with migraine.

## Electronic supplementary material

Below is the link to the electronic supplementary material.

Supplementary material 1 (DOC 154 kb)
