# Supplementary material for: A systematic review of the psychosocial difficulties relevant to patients with migraine
Source: J Headache Pain. 2012 Sep 23;13(8):595–606. doi: 10.1007/s10194-012-0482-1 (PMC3484254; doi:10.1007/s10194-012-0482-1)
Supplement: Supplementary file 1 — Supplementary material 1 (DOC 154 kb) [file 10194_2012_482_MOESM1_ESM.doc]

Appendix I: detailed information on studies’ characteristics

| Reference Number | Author and date | Study type | Sample size | Percentage female | Mean age (SD) | Min-Max age | Disease duration | Migraine with aura | Migraine without aura | Paper quality |
| --- | --- | --- | --- | --- | --- | --- | --- | --- | --- | --- |
| 20 | Framer et al. 2001 | Cross-sectional study | 28 | 100.0% | 43.7 (NA) | 26-64 | NA | NA | NA | 3 |
| 21 | Dasbach et al. 2000 | Randomised trial | 407 | 81.1% | 41 (NA) | 18-65 | NA | NA | NA | 3 |
| 22 | Mulder et al. 2001 | Longitudinal study | 60 | NA | 24.3 (4.9) | NA | NA | 33.3% | 66.7% | 3 |
| 23 | Brandes et al. 2006 | Randomised trial | 468 | 86.8% | 39 (12.1) | NA | NA | NA | NA | 3 |
| 24 | Garcia-Monco et al. 2007 | Controlled clinical trial | 61 | 85.2% | 37 (NA) | 16-56 | NA | 18.0% | 82.0% | 3 |
| 25 | Fuh et al. 2006 | Cross-sectional study | 231 | 81.4% | 35.3 (8.1) | 20-50 | NA | NA | NA | 2 |
| 26 | Freitag et al. 2008 | Randomised trial | 378 | NA | 40.2 (11.1) | NA | NA | NA | NA | 3 |
| 27 | Santanello et al. 2003 | Randomised trial | 1506 | 82.5% | 40.6 (NA) | NA | NA | NA | NA | 4 |
| 28 | Burk et al. 2003 | Longitudinal study | 99 | 88.9% | 43.8 (13.4) | NA | NA | 40.4% | 59.6% | 3 |
| 29 | Bordini et al. 2005 | Longitudinal study | 35 | 91.4% | 40.2 (NA) | 18-60 | NA | 0.0% | 100.0% | 2 |
| 30 | D'Amico et al. 2006 | Longitudinal study | 102 | 86.3% | 36.2 (10.6) | 18-62 | 17.4 | NA | NA | 3 |
| 31 | Lainez et al. 2005 | Longitudinal study | 259 | 67.9% | 39 (8) | 18-61 | NA | 24.7% | 75.3% | 2 |
| 32 | Dahlof et al. 2007 | Randomised trial | 756 | 86.0% | 39.8 (11.5) | NA | NA | NA | NA | 2 |
| 33 | Cady et al. 2008 | Randomised trial | 61 | 85.2% | 42.1 (11.5) | NA | NA | 45.9% | 54.1% | 3 |
| 34 | Gori et al. 2005 | Cross-sectional study | 130 | NA | 38.6 (10.4) | 23-50 | NA | 0.0% | 100.0% | 2 |
| 35 | Lucas et al. 2006 | Cross-sectional study | 10532 | 52.1% | 41.2 (14.5) | NA | 13.2 | NA | NA | 3 |
| 36 | Calandre et al. 2002 | Cross-sectional study | 90 | 56.7% | NA | 18-68 | NA | 16.7% | 83.3% | 3 |
| 37 | Lipton et al. 2000 | Case control study | 729 | 82.6% | NA | 18-65 | NA | NA | NA | 3 |
| 38 | Vasuveda et al. 2003 | Randomised trial | 40 | 87.5% | 38 (NA) | 20-53 | NA | 30.0% | 70.0% | 2 |
| 39 | Pradalier et al. 2004 | Randomised trial | 363 | 80.7% | 39.9 (11.1) | NA | NA | 23.1% | 76.9% | 4 |
| 40 | Vos et al. 2003 | Cross-sectional study | 448 | 84.0% | 46 (NA) | 14-81 | NA | NA | NA | 2 |
| 41 | Nicholson et al. 2005 | Longitudinal study | 21 | 95.2% | 42 (NA) | 22-65 | 21 | NA | NA | 2 |
| 42 | Smith et al. 2007 | Longitudinal study | 565 | 85.8% | 42.3 (11.1) | 18-65 | 20.5 | 26.2% | 73.8% | 2 |
| 43 | Dodick et al. 2009 | Randomised trial | 281 | NA | 38.8 (11) | NA | 20.8 | NA | NA | 4 |
| 44 | Lawler et al. 2006 | Randomised trial | 48 | 83.3% | 41.3 (13.4) | 12-60 | NA | 39.6% | 60.4% | 2 |
| 45 | John et al. 2007 | Randomised trial | 72 | 68.1% | 34.4 (8.7) | NA | NA | 0.0% | 100.0% | 3 |
| 46 | Sculpher et al. 2002 | Controlled clinical trial | 835 | NA | NA | 18-65 | NA | NA | NA | 2 |
| 47 | Lofland et al. 2001 | Longitudinal study | 178 | 89.9% | 39 (NA) | NA | NA | NA | NA | 2 |
| 48 | Silberstein et al. 2007 | Randomised trial | 613 | 40.3% | 39.1 (10.8) | NA | NA | 30.8% | 69.2% | 3 |
| 49 | Diener et al. 2007 | Randomised trial | 514 | 86.6% | 40.1 (10.6) | 18-80 | NA | NA | NA | 4 |
| 50 | Schellenberg et al. 2008 | Randomised trial | 30 | 86.7% | 39 (10) | NA | 17 | 96.7% | 3.3% | 2 |
| 51 | Guyron et al. 2005 | Randomised trial | 108 | NA | 43.4 (NA) | NA | NA | 25.9% | 74.1% | 2 |
| 52 | Guyron et al. 2009 | Randomised trial | 75 | NA | 44.9 (NA) | 26-76 | NA | 38.7% | 61.3% | 3 |
| 53 | Lee et al. 2005 | Randomised trial | 74 | 87.8% | 42.6 (14) | NA | NA | 0.0% | 100.0% | 2 |
| 54 | Dowson et al. 2007 | Longitudinal study | 28 | 85.4% | 44.7 (NA) | NA | NA | NA | NA | 2 |
| 55 | Diamond et al. 2005 | Randomised trial | 756 | 85.0% | NA | 12-70 | NA | NA | NA | 3 |
| 56 | Millan-Guerrero et al. 2008 | Randomised trial | 90 | 85.6% | 32.8 (9.9) | NA | 14 | 8.9% | 91.1% | 3 |
| 57 | Narin et al. 2003 | Controlled clinical trial | 40 | 100.0% | 35.2 (10.2) | NA | NA | 0.0% | 100.0% | 2 |
| 58 | Rigatelli et al. 2009 | Controlled clinical trial | 20 | 60.0% | 35 (6.7) | NA | NA | 100.0% | 0.0% | 2 |
| 59 | Merelle et al. 2008 | Controlled clinical trial | 95 | 86.3% | 44 (NA) | 24-63 | NA | 34.7% | 65.3% | 3 |
| 60 | Lemstra et al. 2002 | Randomised trial | 80 | 66.2% | 35.6 (10.1) | NA | 8.6 | NA | NA | 3 |
| 61 | Stronks et al. 2003 | Randomised trial | 12 | NA | 42.2 (9.8) | 20-59 | NA | 8.3% | 91.7% | 3 |
| 62 | Wacogne et al. 2003 | Cross-sectional study | 250 | 71.2% | 38.2 (12.4) | NA | 20 | NA | NA | 3 |
| 63 | Tronvik et al. 2003 | Randomised trial | 57 | 78.9% | 42 (11) | NA | NA | NA | NA | 4 |
| 64 | Frediani et al. 2004 | Cross-sectional study | 1810 | 100.0% | NA | 18-35 | NA | NA | NA | 2 |
| 65 | Holroyd et al. 2007 | Longitudinal study | 232 | 79.0% | 38 (NA) | NA | 15 | NA | NA | 3 |
| 66 | Barbanti et al. 2007 | Case control study | 200 | 84.0% | 36.9 (12.1) | NA | 18.9 | 14.0% | 86.0% | 3 |
| 67 | Millan-Guerrero et al. 2007 | Randomised trial | 92 | NA | 32.6 (9.8) | NA | 17.4 | 10.9% | 89.1% | 2 |
| 68 | Park et al. 2008 | Cross-sectional study | 92 | 81.5% | 36.6 (9.5) | NA | NA | 0.0% | 100.0% | 2 |
| 69 | Geraud et al. 2008 | Longitudinal study | 5417 | 80.0% | 41.4 (12.4) | 18-88 | 10.9 | 0.0% | 100.0% | 3 |
| 70 | Varkey et al. 2009 | Longitudinal study | 20 | 85.0% | NA | 36-73 | 32 | 60.0% | 40.0% | 2 |

NA: the information was not available
